# Craniocervical Myology and Functional Morphology of the Small-Headed Therizinosaurian Theropods *Falcarius utahensis* and *Nothronychus mckinleyi*

**DOI:** 10.1371/journal.pone.0117281

**Published:** 2015-02-23

**Authors:** David K. Smith

**Affiliations:** Department of Biology, Northland Pioneer College, Holbrook, Arizona, United States of America; State Natural History Museum, GERMANY

## Abstract

Therizinosaurs represent a highly unusual clade of herbivorous theropods from the Cretaceous of North America and Asia. Following descriptions of the basicrania of the North American therizinosaurs *Falcarius utahenisis* and *Nothronychus mckinleyi*, the craniocervical musculature in both taxa is reconstructed using *Tyrannosaurus*, *Allosaurus*, and some extant birds as models. These muscles are subdivided into functional groups as dorsiflexors, lateroflexors, and ventroflexors. Lateroflexors and dorsiflexors in *Nothronychus*, but not *Falcarius*, are reduced, from the plesiomorphic theropod condition, but are still well developed. Attachments in both genera are favorable for an increase in ventroflexion in feeding, convergent with *Allosaurus fragilis*. *Falcarius* and *Nothronychus* are both characterized by a flat occipital condyle, followed by centra with shallow articular facets suggesting neck function very similar to that of an ostrich *Struthio camelus*. Neck movement was a combined result of minimal movement between the individual cervical vertebrae.

## Introduction

**Fig 1:**
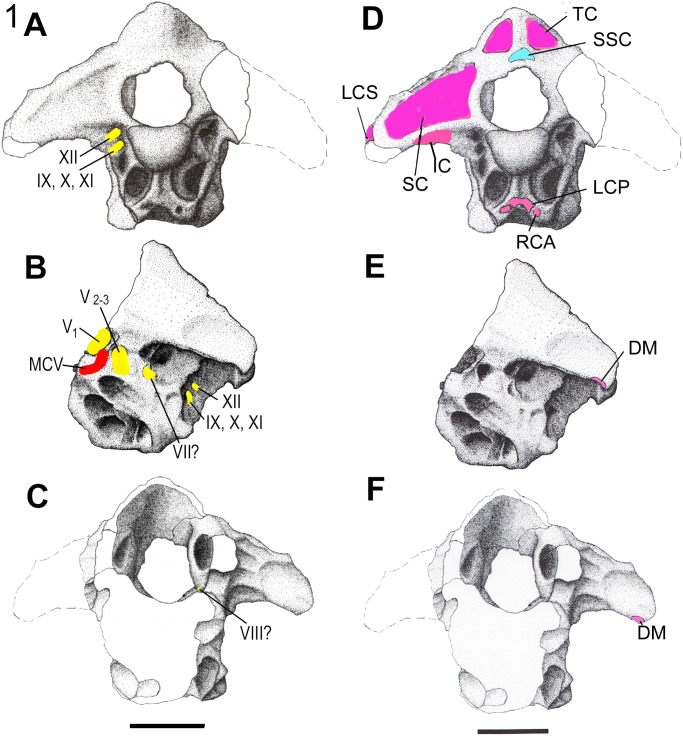
The braincase of *Falcarius utahensis* (UMNH VP 15000), Barremian, Lower Cretaceous Cedar Mountain Formation, Crystal Geyser Site, Utah in A, posterior; B, left lateral; and C, anterior views. Reconstructed nerves are indicated in yellow and blood vessels in red. The braincase of *Falcarius* in **D**, posterior; **E**, left lateral; and **F**, anterior views. Reconstructed tendon attachment points are indicated in blue and muscle insertion points in pink. The scale bar equals approximately 2 centimeters. Modified from Smith et al. [11].

**Fig 2:**
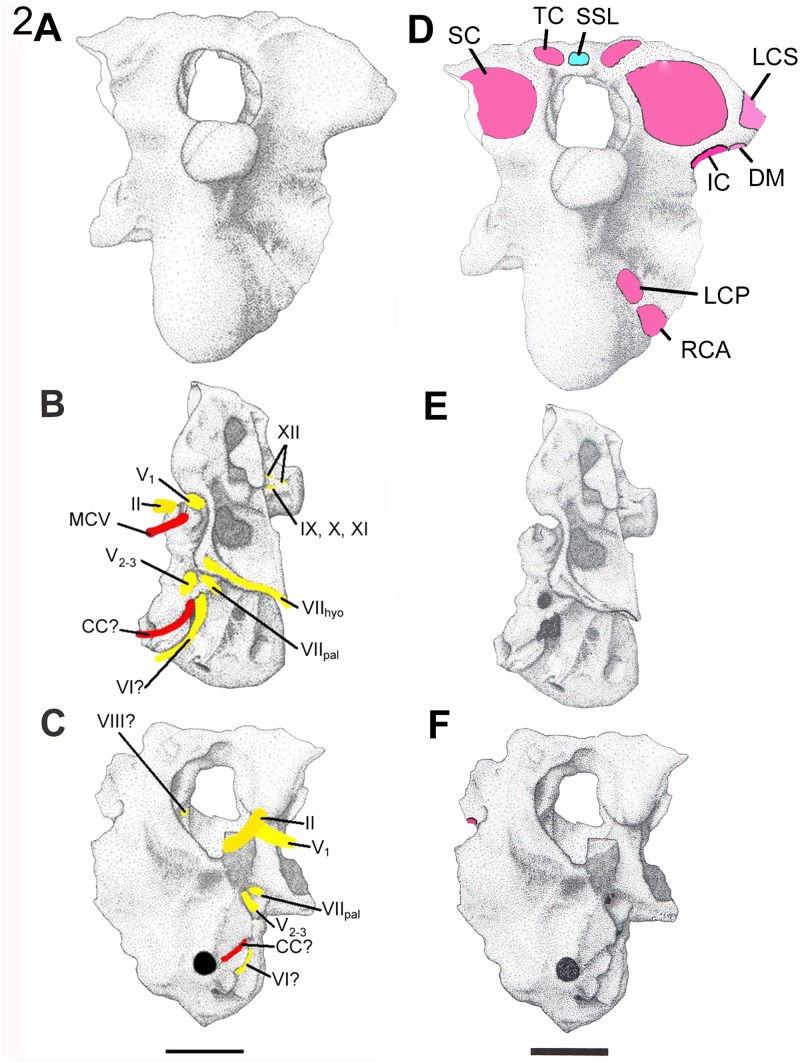
The braincase of *Nothronychus mckinleyi* (AzMNH P-2117), Turonian, Lower Cretaceous Moreno Hill Formation, west-central New Mexico in A, posterior; B, left lateral; and C, anterior views. Reconstructed nerves are indicated in yellow and blood vessels in red. The braincase of *Nothronychus* in **D**, posterior; **E**, left lateral; and **F**, anterior views. Reconstructed tendon attachment points are indicated in blue and muscle insertion points in pink. The scale bar equals approximately 2 centimeters. Modified from Smith [12].

Reconstructions of craniocervical musculature are regarded as being increasingly important in understanding feeding behavior in extinct vertebrates. Most of the recent work has focused on hypercarnivorous large headed theropods including *Ceratosaurus nasicornis*, *Allosaurus fragilis*, and the tyrannosaurids *Albertosaurus sarcophagus* and *Tyrannosaurus rex* [1–4]. Jaw muscles and function in such selection of theropods have been reconstructed [5–8]. Small headed theropods, however, have received considerably less attention from a myological perspective. An initial hypothesis would be that craniocervical musculature of small headed coelurosaurs was relatively lighter than in their larger-headed relatives, especially with shifts to herbivory [9, 10] because less force and torque is required to maneuver a relatively light skull. For example, the basicranium of derived therizinosaurian herbivores (Figs. 1, 2), such as *Nothronychus mckinleyi*, were extensively modified from their basal condition in *Falcarius utahensis* and the plesiomorphic theropod condition [11, 12]. The craniocervical musculature and resulting lever arms were subjected to some changes, as well.

Cervicocephalic muscles insert on the basicranium, which has recently been described for the therizinosaurs *Falcarius* and *Nothronychus* [11, 12]. Here, a reconstruction of the major craniocervical muscles for *Falcarius* and *Nothronychus* is reported, using supplemental information for *Erlikosaurus andrewsi* [13, 8] along with nervous and vascular reconstructions (Figs. 1, 2). Following past studies [14, 3], the size of each muscle in this region is assumed to be correlated with the size and rugosity of their attachment points. For example, the size of the epaxial muscles is taken to be a function of the height of the neural arch plus the spine, as evident in lariform birds and proposed for larger theropods [2]. Osteological inferences follow recent therizinosaurian literature [11, 12] and cervical muscle terminology follows recent descriptions [2. 3] and Tsuihiji [4]. Since the therizinosaurian braincase closely resembles the avian condition, avian terminology is retained, where possible, but homologies with crocodilian musculature are noted.

## Methods and Materials

### Specimen Repository

No permits were required for the described study, which complied with all relevant regulations. The *Nothronychus* braincase and associated vertebrae (AzMNH 2117) (Arizona Museum of Natural History, Mesa, Arizona) were collected from the Turonian Moreno Hills Formation, Zuni Basin, New Mexico. The *Falcarius* braincase and vertebrae were collected from the Barremian Cedar Mountain Formation, Crystal Geyser, Utah. Both braincases have previously been described [11, 12]. The original specimens are stored at the Arizona Museum of Natural History, Mesa, Arizona (AzMNH 2117) and Utah Museum of Natural History, Salt Lake City, Utah (UMNH 15000). Casts are available at the Museum of Northern Arizona, Flagstaff, Arizona.

### Soft Tissue Reconstruction

The *Falcarius* and *Nothronychus* basicrania are roughly the same size. Muscle attachment points were identified for the occiput and cervical vertebrae (Figs. 1, 2; Table 1) following those proposed for other theropods [2–4]. The posterior braincase and a number of cervical vertebrae in *Nothronychus* were probably all derived from a single individual (McCord, pers. comm., 2013). Otherwise, information for the therizinosaurian cervical vertebrae had to be gathered from published information a number of genera, including *Jianchangosaurus yixianensis*, *Neimongosaurus yangi*, and *Falcarius utahensis*, along with specimens of *Nothronychus utahensis*. In support of this muscular reconstruction, the currently available *Nothronychus* cervical vertebrae are discussed. Following the extant phylogenetic bracketing approach [15], the occiput of *Nothronychus* was compared with casts of skulls from the extant Tuatara (*Sphenodon punctatus*), Water monitor (*Varanus salvator*), Caiman (*Paleosuchus trigonatus*), Pigeon (*Columba livia*), and Emu (*Dromaius novaehollandiae*) skulls. The craniocervical region of a pigeon was dissected following Chiasson [16].

**Table 1:** Therizinosaur craniocervical muscle origins, insertions, and functions based on theropod inferences of Snively and Russell [2, 3] and Tsuihiji [4].

Muscle	Origin	Insertion	Function
Transversospinalis capitis	Cervical Neural Spines	Supraoccipital, Parietal	Dorsiflexing Head
Splenius Capitis	Cervical #2 Neural Spine	Supraoccipital, Parietal	Lateroflexing Head
Longissimus Capitis Superficialis	Cervical Parapophyses, Neural Arches	Distal Paroccipital Processes	Lateroflexing, Ventroflexing (?) Head
Iliocostalis Capitis	Cervical Hypopophyses or Centra	Distal Paroccipital Processes	Lateroflexing, Ventroflexing (?) Head
Longissimus Capitis Profundus	Cervical Transverse Processes	Basioccipital	Ventroflexing Head
Rectus Capitis Anterior	Ventral Cervical Vertebrae	Basal Tubera	Ventroflexing Head
Depressor Mandibulae	Distal Paroccipital Processes, Squamosal	Retroarticular Processes	Depressing Mandible

The epaxial muscles originate from the neural spines and neural arches of the cervical vertebrae, so they have been used to index their relative development [2]. Tyrannosaurs are characterized by large, well-developed neural spines [2]. A nearly complete to complete cervical series for most therizinosaurs is not available, except for *Neimongosaurus* [17], *Nanshiungosaurus* [18] and *Jianchangosaurus* [19]. Two posterior cervical vertebrae are attributed to *Erliansaurus* [20] and three for the holotype of *Beipiaosaurus* [21]. A second specimen referred to *Beipiaosaurus* [22] possesses a complete cervical series consisting of 11 vertebrae. They are not described, but appear quite morphologically uniform in the figure. Some isolated cervical vertebrae were described for *Falcarius utahensis* [23]. A few distorted cervical vertebrae are known for *Nothronychus mckinleyi* [24]. Where known and described, most therizinosaurs possess short, small neural spines in the cervical vertebrae, similar to those of ratites [25], and small occiputs. Cervical morphology in *Neimongosaurus* is fairly consistent, varying mostly in size [17]. In *Falcarius*, the anterior and mid-series centra are described as antero-ventrally flexed and the neural spines are anteroposteriorly elongate, but dorsally reduced to small ridges [23]. Relative heights of the neural spines above the centra are consistent for the available cervical vertebrae for all of the known therizinosaurian genera. It is currently difficult to obtain an exact ratio between the heights of the neural spine to total neck length for the North American therizinosaurs, as was done for tyrannosaurs and *Allosaurus* [2], but the absolute and relative neural spine heights of the therizinosaurs were very short relative to the total neck length [17–24].

The occipital condyle of *Erlikosaurus* is more spherical and the supraoccipital more vertically oriented than in *Falcarius* or *Nothronychus* [11–13], both characters likely correlated with increased mobility at that joint and increased moment arms for the epaxial musculature. As in theropods, generally, the neck of therizinosaurians was probably S-shaped [2]. This hypothesis would be supported by the completely horizontally directed occipital condyle in all three known braincases.

As in any animal, the musculature would be heavily influenced by the surrounding bone and soft tissue, including nerves, blood vessels, and tendons. Foramina transmitting the optic (II), trigeminal (V), facial (VII), vestibulocochlear (VIII), glossopharyngeal (IX), vagus (X), spinal accessory (XI), and hypoglossal (XII) cranial nerves are partially to completely preserved. Some nerves shared common canals. The endocranial cavity of the therizinosaurs [25, 11, 12] is quite similar to that of *Troodon* [26]. The cerebellar fossa has an intermediate morphology between a peaked and more rounded form observed in two specimens of *Troodon*. As expected, the optic tectum for *Falcarius* and *Nothronychus* was probably laterally oriented as in other coelurosaurs [27]. The location of the optic nerve constrains the size of the adductor chamber in both taxa. The chamber is reduced, resulting in reduced adductor musculature and associated bite forces [12].

The anterior middle cerebral vein (MCV) is interpreted as exiting through a groove at the posterior margin of the trigeminal foramen in *Falcarius* [11] and through a notch at the base of the same foramen in *Nothronychus* [12]. In both genera, the venous canal is included with the foramen. This location would be plesiomorphic for saurischians [27–29]. In derived theropods, it is contained within the laterosphenoid anterior to the trigeminal foramen [27–29], so this placement represents an unexpected reversal in these therizinosaurs. The recovery of a complete laterosphenoid for *Nothronychus* and *Falcarius* will alleviate this question. The carotid canal (CC) is interpreted as contained within a groove in *Nothronychus*.

### Muscle Reconstruction

#### M. transversospinalis capitis

M. transversospinalis capitis (TC) originates at the tips of the cervical neural spines and probably inserts at the dorsal margin of the parietal, at least in large theropods [2]. Snively and Russell [2] use the term m. transversospinalis capitis, which is homologous with m. biventer cervicis in birds, with a separate m. complexus for tyrannosaurs. Tsuihiji [30, 4] retained a proposed single two-headed m. transversospinalis capitis with a medial head and a lateral head in Crocodylia, but split it into two muscles, a medial portion, m. spinalis capitis (lepidosaurs) or m. biventer cervicis and m. longus colli dorsalis, pars caudalis (avian) inserting on the supraoccipital and parietal, and a lateral portion, m. longissimus capitis, pars articuloparietalis (lepidosaurs) or m. complexus (avian) inserting on the squamosal and distal end of the paroccipital process. In extant pigeons, Chiasson [16] described both muscles as inserting at the lambdoidal crest on either side of the supraoccipital (cerebellar prominence), m. biventer cervicis deep to m. complexus and this relationship was observed in a dissected specimen.

In large theropods, it originated from the tips of the cervical neural spines [2–3]. As the neural spines are much shorter in the known cervical vertebrae of therizinosaurs than tyrannosaurs, anterior origins of m. transversospinalis capitis could not have been much higher than the occipital condyle (similar to the condition in birds [2–4]). In birds, this muscle is quite slender [2] and terminates in a long tendon and small muscle belly at the occiput in pigeons [16] and the ostrich *Struthio camelus* [3].

M. transversospinalis capitis [2, 3] medially inserted on the supraoccipital and possibly the parietal on either side of the supraspinous ligament (SSL). The supraocipital and parietal, however, are much higher in tyrannosaurs than in *Falcarius* or *Nothronychus*. The ligament attachment point is represented by a shallow excavation at the base of the supraoccipital, reduced to a small medial knob, dorsal to the foramen magnum in *Falcarius*. M. transversospinalis capitis insertion points are shallow excavations lateral to the crest, as has been reconstructed in other theropods [4] as m. spinalis capitis. The supraspinous ligament attachment point is reduced even further in *Nothronychus* to a very shallow excavation dorsal to the foramen magnum. The insertion points are correspondingly faint, but can be determined.

In tyrannosaurs, Snively and Russell [2] regard this muscle as more similar to m. transversospinalis capitis of crocodilians than m. biventer cervicis described in birds, but with a more dorsal parietal insertion than in extant archosaurs. In these theropods, the dorsally concave curve flexure of the neck is pronounced, resulting in a ventral concavity. Snively and Russell [2] infer that m. transversospinalis capitis filled this concavity. In maniraptoran theropods, however, this muscle has reduced origins and insertions, so it is suggested that the homologous m. biventer cervicis is more similar to the common avian condition, where it extends within fascia associated with the neck dorsiflexor m. longus colli dorsalis [2]. These muscles and fascia roughly follow the posterior concavity of the neck, smoothing it out in tyrannosaurs and allosaurs [2, 3]. Given the relatively low neural spines in therizinosaurs, however, this development was probably reduced, resulting in a relatively narrow neck similar to that observed in ostriches.

Snively and Russell [3] describe the major function of m. transversospinalis capitis/biventer cervicis as dorsiflexing the head relative to the cervicals in birds and crocodilians, which would occur with inertial feeding in both taxa and in opening the jaws in crocodilians. Similar activities are supported for tyrannosaurs [3]. This function is inferred for *Falcarius* and *Nothronychus*, but the insertion for this muscle is better developed and associated with a longer lever arm, but still weak, in *Falcarius*. This development suggests that the dorsiflexive function was probably weak and might have been partially subsumed by increased arm mobility and the neck moving as a unit in *Falcarius* and more so in *Nothronychus*.

#### M. complexus

In pigeons, m. complexus (C) is a broad, thin muscle that originates from the transverse processes of the third and fourth cervical vertebrae [16], but in some birds it also arises from the epiphyses anterodorsolateral to the insertion of the m. longus colli dorsalis [2, 3]. This muscle is equivalent to m. longissimus capitis, pars articuloparietalis in lepidosaurs and the lateral head of the m. transversospinalis capitis in Crocodylia [4]. It is reconstructed as arising from the epiphyses of all of the anterior cervical vertebrae in tyrannosaurs [2], as in some birds [31, 2]. In these animals, the zygapophyses of the cervical vertebrae would interfere with the course of the muscle from the transverse processes to its dorsal insertion on the occiput (Snively, pers. com. 2013). However, in therizinosaurs, as in pigeons, there is no such interference as the zygapophyses and epipophyses are relatively small (Snively, pers. com., 2013). In *Falcarius*, and probably *Nothronychus*, the structures referred to the postzygadiapophyseal laminae [23] probably represent origination points for m. complexus. These were apparently modified transverse processes that were deflected ventrally. They are reconstructed here as origination sites for the m. complexus.

M. complexus inserts on the medial squamosal and lateral lambdoidal crest of the parietal on either side of the cerebellar prominence lateral to the m. biventer cervicis insertion in pigeons [16]. Snively and Russell [3] describe the m. complexus insertion as on the same points in the squamosal and parietal, lateral to the foramen magnum in tyrannosaurs. In *Erlikosaurus*, the insertion point was probably at homologous points, placing it lateral and slightly dorsal to the foramen magnum [13]. The m. complexus insertion was probably similar for both *Falcarius* and *Nothronychus*, dorsolateral to the foramen magnum. In neither latter case, however, is the squamosal preserved. Tsuihiji [4] regards the muscle, in his discussion of m. longissimus capitis pars articuloparietalis, as inserting broadly, and more ventrally, on the parietal crest in tyrannosaurs, but these were considerably larger animals with larger skulls than all known therizinosaurs. In one of several alternate reconstructions, Snively and Russell [2, 3, 32] posit the muscle as inserting more broadly on the occipital region of tyrannosaurs, including on the squamosals medial to the origin of m. depressor mandibulae.

Snively and Russell [3] describe m. complexus as active in dorsiflexion and lateroflexion in birds, so is, at least in part, synergistic with m. biventer cervicis, partially contributing to inertial feeding. In crocodilians, it is more important in lateroflexion than dorsiflexion. As noted by Snively and Russell [3], the tyrannosaurid skull is taller than in crocodilians, giving the m. complexus a stronger dorsiflexive lever arm. Conversely, lateroflexion of the head by this muscle was weaker in *Tyrannosaurus* and *Albertosaurus* than in many birds; electromyography confirms this function in chickens [33]. The proposed insertion of m. complexus in therizinosaurs would result in a further reduced contribution of this muscle to dorsiflexion and increased activity during lateroflexion, similar to its function in crocodilians.

#### M. splenius capitis (m. epistropheo-capitis and m. altoido-capitis)

The avian medial splenius capitis is regarded as homologous with m. altoido-capitis and laterally with m. epistropheo-capitis in Crocodylia and with m. rectus capitis posterior and m. obliquus capitis magnus in lepidosaurs [4]. In crocodiles and birds, this muscle originates on the neural spine of the second cervical vertebra and inserts on the paroccipital process [3], but Snively and Russell describe the medial portion as shifted onto the parietal in tyrannosaurs and the lateral portion as inserting on the squamosal in albertosaurs. Tsuihiji [4] describes the medial insertion as located on the supraoccipital (as m. rectus capitis posterior) in lepidosaurs, crocodiles, and birds.

Unfortunately, neither the parietal nor the squamosal is preserved for *Falcarius* or *Nothronychus*. In *Erlikosaurus*, the dorsal margin of the parietal forms a modest parietal crest [13]. The posterior surface of the parietal is concave, with the crest oriented anteromedially in dorsal view. Snively and Russell [3] describe a medial head of the m. splenius capitis as inserting on this surface in albertosaurs, with a resulting strong associated dorsiflexive moment arm. The corresponding attachment would have been relatively weaker in *Erlikosaurus*.

The medial process of the squamosal is described as overlapping the parietal and overlying the distal end of the paroccipital process [13] The posterior portion of the parietal and squamosal combined form a small notch above the paroccipital process, broadly similar to, but apparently less well-defined, than in tyrannosaurs [34, 3]. As in albertosaurs, a lateral head of the splenius capitis may have inserted here in *Erlikosaurus*, with a weak contribution to lateroflexive movement [3].

In both *Falcarius* and *Nothronychus*, the posterior surface of the paroccipital process is broadly concave, reflecting an insertion for a large muscle. Tsuihiji [4] regards this region as the insertion for m. splenius capitis. In *Falcarius*, the ventral margin is interpreted as a faint medial ridge extending from the dorsal surface of the occipital condyle. *Nothronychus* is similar, but the ridge is fainter and extends from the occipital condyle to the base of the ventral margin of the paroccipital process, resulting in a dorsoventrally broader insertion.

In both North American therizinosaurs, m. splenius capitis would have had a lateroflexive function. This function appears to have been broadly similar in *Erlikosaurus*, based on the description of the skull by Clark [13].

#### M. longissimus capitis superficialis

Tsuihiji [4] regards m. longissimus capitis superficialis (LCS) as equivalent to m. longissimus capitis, pars transversalis capitis in lepidosaurs and absent in birds. In Tsuihiji’s reconstruction for *Daspletosaurus torosus*, based solely on the hypothesized insertion point on the distal end of the paroccipital process, it appears to be a combination of the avian m. rectus capitis lateralis and possibly the lepidosaurian m. episternocleidomastoideus, but the latter muscle has a separate origination on the shoulder girdle and sternum distinct from the former [4].

In crocodilians, m. longissimus capitis superficialis originates from parapophyses and the ventrolateral neural arches of the fifth through ninth neural arches [3]. Snively and Russell [2] report the presence of proximal scars on the parapophyses of the seventh through ninth cervical vertebrae in tyrannosaurs, but not four through six.

M. longissimus capitis superficialis inserts at the lateral portion of the paroccipital process in crocodilians [2, 3]. They indicate that the insertion was probably similar in large theropods. The distal ends of these processes are quite broad in tyrannosaurs [29, 2] compared to their development in *Falcarius* and *Nothronychus*, where they are short and small [10, 11]. The distal end of the paroccipital process is somewhat rugose in *Nothronychus*, whereas there is a distinct cavity in the posteroventral surface of the distal end of *Falcarius*. In *Nothronychus*, this surface probably served as a combined attachment point for m. longissimus capitis superficialis and m. depressor mandibulae. The cavity in *Falcarius* is proposed as the insertion for m. longissimus capitis superficialis, with the m. depressor mandibulae attaching at a separate, anteroventrally oriented excavation.

M. longissimus capitis superficialis strongly contributes to lateroflexion of the head in crocodilians and, presumably in tyrannosaurs. In both *Allosaurus* and *Falcarius*, but not *Nothronychus* or other theropods, m. longissimus capitis superficialis would have inserted ventrolateral to the occipital condyle (Figs. 1, 2), resulting in a ventroflexive action for the head with contraction [1, 2].

#### M. iliocostalis capitis

M. iliocostalis capitis (IC) in crocodilians was considered homologous with m. rectus capitis lateralis in birds [2]. In birds, it originates from either the lateral hypopophysis or the ventrolateral corner of the centrum of the second cervical vertebra. In the pigeon, m. rectus capitis lateralis originates on “ventral” processes of some cervical vertebrae [16]. This attachment is limited to the ends of diapophyses of cervical vertebrae three, four, and five in the raven [35] but Snively and Russell [3] limit the origin to either the hypapophysis of the second cervical vertebra, posterior to m. rectus capitis anterior or from the ventrolateral corner of the centrum in the crow (*Corvus brachyrhynchus*). In crocodilians, a likely homology of m. iliocostalis costalis originates from fascia about the first and second cervical ribs [2]. Snively and Russell [3] regard the origin of m. iliocostalis capitis as unclear in tyrannosaurs. They skeptically suggest that it may have originated from the heads of the cervical ribs.

Tsuihiji [4] differentiates the muscle originating at the anterior cervical ribs from others in the iliocostalis series, demonstrating that it inserts on the lateroventral end of the paroccipital process. It is reconstructed for tyrannosaurs by Tsuihiji [4] as inserting at the distal ends of the paroccipital processes. Here, the large muscle inserting along the posterior surface of the paroccipital process is referred to m. obiquus capitis magnus in lepidosaurs, which would be homologous with the avian lateral m. splenius capitis.

M. iliocostalis capitis probably inserted on the ventral margin of the paroccipital process in both *Falcarius* and *Nothronychus* ventral to the m. splenius capitis insertion. This position would result in a lateroflexive function in *Nothronychus*, but some additional ventroflexive component in *Falcarius*.

In most archosaurs, m. iliocostalis capitis lateroflexes the head and contributes to rotation of the head [3]. In crocodilians, it may be antagonistic to the dorsiflexors during elevation. In tyrannosaurs and most other theropods [1, 2], including *Nothronychus*, the paroccipital processes extend laterally from the foramen magnum. Therefore, m. iliocostalis capitis functions primarily as a lateroflexor of the head. In *Falcarius*, however, the paroccipital processes are deflected ventrally (Figs. 1, 2) as they are in *Allosaurus* [1], which would result in ventroflexion by this muscle.

#### M. longissimus capitis profundus

Tsuihiji [4] describes m. longissimus capitis profundus (LCP) referred to by Snively and Russell [2] as homologous with the combination of m. longissimus capitis, pars transversalis cervicis and m. iliocostalis capitis. In both recent studies, Tsuihiji [4] notes its relationship with m. rectus capitis anterior as reversed from the proposed reconstruction in earlier studies on basal ornithopods and tyrannosaurs [36–38]. M. longissimus capitis profundus is described as originating from the lateral sides of the transverse processes of the cervical vertebrae and inserting on the basioccipital ventral to the occipital condyle in birds and crocodilians [3]. The attachment points for the proposed combination of muscles described by Tsuihiji [4] are more complex. M. longissimus capitis, pars transversalis cervicis originates on the lateral surfaces of the neural arches and transverse processes and inserts on the basal tubera in lepidosaurs and crocodilians. However, m. iliocostalis capitis, pars transversalis cervicis originates in fascia within the neck and inserts on the basal tubera in lepidosaurs. In crocodilians, the muscle, combined with m. rectus capitis lateralis and m. episternocleidomastoideus, originates at the lateral side of the atlas rib and inserts on the distal end of the paroccipital process [39, 4]. In birds, m. longissimus capitis pars transversalis cervicis and m. iliocostalis capitis are combined into one muscle that Tsuihiji [39] calls m. rectus capitis dorsalis. This combined muscle inserts on the basal tubera [4]. These different architectures, however, should not affect the functional aspects of the current discussion. The muscle, or combination of muscles, would contribute to ventroflexion of the head, as described by Snively and Russell [3].

Snively and Russell [2] reconstruct m. longissimus capitis profundus as originating from the transverse processes of the anteriormost cervical vertebrae in tyrannosaurs. Both Snively and Russell [2] and Tsuihiji [4] reconstruct this muscle as inserting on the proximal basal tubera or condylotuberal crest ventral to the occipital condyle in a pronounced excavation in tyrannosaurs. This attachment would presumably subdivide an external pneumatic cavity in tyrannosaurs posterior to the basioccipital/basisphenoid as described by Currie [40] and Tsuihiji [4].

The cervical transverse processes are reduced in *Neimongosaurus* [17], *Falcarius*, and probably all other therizinosaurs, so this muscle is also regarded as reduced (Figs. 1, 2). *Falcarius* possesses a pronounced condylotuberal crest descending from the occipital condyle [11] that subdivides a deep pneumatic chamber as in tyrannosaurs and presumably serves as the insertion for m. longissimus capitis profundus. In this species of *Nothronychus*, the crest and associated basal tubera are reduced and form a dorsolateral margin of an enclosed bulla [12]. The posterior pneumatic cavities are represented by shallow, posteriorly directed excavations lateral to the bulla.

#### M. rectus capitis anterior

Tsuihiji [4] regards m. rectus capitis anterior (RCA) of lepidosaurs as homologous with m. rectus capitis anticus major (Crocodylia) and m. rectus capitis anterior (Aves). Snively and Russell [2] use the avian terminology in this case. Snively and Russell [3] describe the origination of m. rectus capitis anterior (as m. rectus capitis ventralis) as the ventral surfaces of the anterior cervical vertebrae in birds and crocodilians. In lepidosaurs and crocodilians, it passes medial to the cervical ribs and inserts in the distal basal tubera below the insertion for m. longissimus capitis profundus (m. longissimus capitis, pars transversalis cervicis and m. iliocostalis capitis of Tsuihiji [2] and Snively and Russell [4].

M. rectus capitis anterior is reconstructed as originating at the hypopophyses and adjacent cervical centra and inserts in pronounced pits in the basal tubera in tyrannosaurs [3], although these depressions may be fossae pneumatized by the paratympanic sinus system [26]. This muscle is closely associated with ventroflexion.

M. rectus capitis anterior inserts in a small posteriorly directed pit in the distal basal tubera in *Falcarius* [11]. This location would make it ventral to the deep pneumatic pit. In *Nothronychus*, the insertion point and the associated pneumatic pit is less well-defined and so is taken as the region of faint basal tubera distal to the condylotuberal crest. Its function in the two therizinosaurs would be cranial ventroflexion, as in tyrannosaurs.

#### M. depressor mandibulae

In the emu, the posterior wall of the squamosal bears a clear excavation for the origin of m. depressor mandibulae (DM). The scar continues onto the distal end of the paroccipital process. In the caiman, the origin for this muscle is mainly in the distal paroccipital process, with a reduced squamosal origin. Tsuihiji [4] reconstructed the origin of m. depressor mandibulae in tyrannosaurids as including both elements. The posteroventral margin of the distal paroccipital processes of *Falcarius* and *Nothronychus* are rugose and slightly expanded. These scars are interpreted as the partial origin of m. depressor mandibulae in these therizinosaurs (Figs. 1, 2). The squamosals of both *Falcarius* and *Nothronychus* are currently unknown, but the m. depressor mandibulae origin certainly included both elements [23, 4].

M. depressor mandibulae inserts on the retroarticular process of the mandible in alligators and pigeons and certainly did so in *Falcarius utahensis* and *Nothronychus mckinleyi*, as in other dinosaurs [7], but the posterior portion of the mandible is not preserved for these taxa. Presumably, the retroarticular process is similar to that of *Erlikosaurus* [13].

## Conclusions

**Fig 3:**
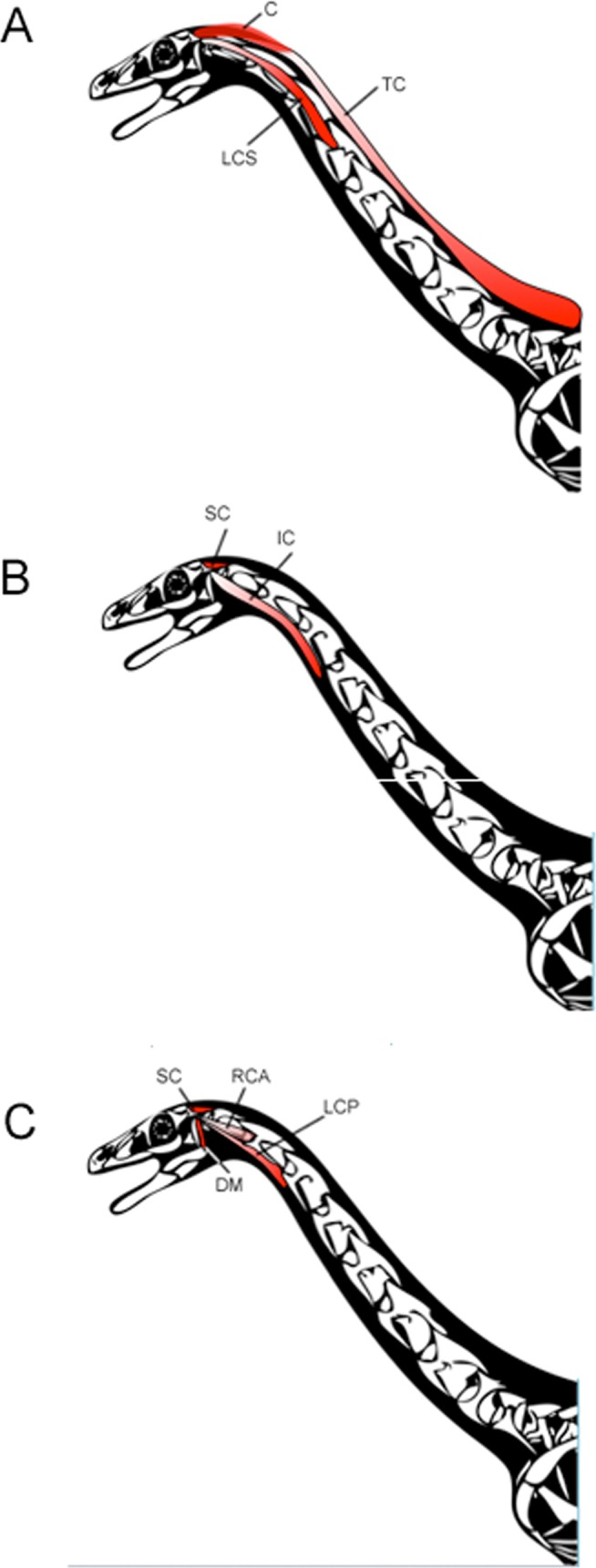
Reconstructed craniocervical musculature in the therizinosaurs *Falcarius utahensis* and *Nothronychus mckinleyi* (after Scott Hartman, 2013). **A**, superficial; **B**, middle; and **C**, deep musculature. In the deep musculature, m. rectus capitis anterior would pass medial to the cervical ribs. Modifications by Eric Snively and David Smith.

Hypothesized therizinosaur function is deduced from the presented craniocervical muscle reconstructions (Fig. 3) and compared to other theropods. The neck muscle function for tyrannosaurids has been extensively studied by Snively and Russell [2, 3, 32] and Tsuihiji [4] and their results can be applied here (Table 1), with suitable alteration for the differing osteologies. Tyrannosaurs and many other large theropods were characterized by a tall, broad nuchal crest reflecting an increased insertion for the epaxial musculature and associated leverage for cranial dorsiflexion [2]. This development is reduced in the known therizinosaurs *Falcarius* and probably *Nothronychus*. The posterior surface of the basal tubera in *Falcarius*, but not *Nothronychus*, is quite rugose, suggesting a reduction in the ventral flexors between the genera. The positions of the tubera are anteriorly equivalent in the two taxa, but those of *Nothronychus* are much more widely spaced [12], paralleling variation in cervical mechanics as seen in different tyrannosaurs [32, 2].

Both *Falcarius* and *Nothronychus* emphasized lateroflexion. The paroccipital processes of *Falcarius* are much longer, with a greater posteroventral orientation than *Nothronychus*. Therefore, the paroccipital processes in *Nothronychus* have reverted to a lateral orientation, as is typically the case in theropods. The resulting craniocervical movement was a combination here, as in theropods primitively, of m. longissimus capitis superficialis, m. intercostalis capitis, and m. splenius capitis [2]. In *Falcarius*, especially, there was probably some contribution by m. complexus. The resulting lever arm for both of these muscles is more laterally extensive in *Falcarius* than *Nothronychus*. This pattern suggests a reduction in the relative importance, at least of these muscles, in *Nothronychus* compared to *Falcarius*, but in both genera, lateroflexion was more important than dorsiflexion or ventroflexion.

Increased ventroflexion over dorsiflexion was probably present in both *Falcarius* and *Nothronychus*, but was accomplished in different ways. In *Falcarius*, but not *Nothronychus*, there is an unusual ventral deflection of the paroccipital processes such that the ends extend ventrally to the level of the base of the occipital condyle. This orientation is even more exaggerated in the braincase of *Allosaurus*, where the distal ends extend ventral to the condyle [41, 1, 2]. Therefore, m. iliocostalis capitis and m. longissimus capitis superficialis may have had some ventroflexive component, as well as countering dorsiflexion, in *Falcarius*, but not as extreme as in *Allosaurus* [2, 41]. In *Allosaurus*, this pattern is associated with a change in the lever arms, increasing the co-opting these muscles for increased ventroflexive torque [2, 3, 42]. Bakker [1] proposed that allosaurids increased the ventroflexing craniocervical muscles, while reducing the temporal region, so that they acquired more participation of the neck muscles while reducing the contribution of direct bite forces.

Ventroflexion was primarily accomplished in *Nothronychus* and *Falcarius* through a ventral displacement of m. rectus capitis anterior and m. longissimus capitis profundus, with a resulting long lever arm. In case of either of these therizinosaurs, there may have been a notable downward stroke during feeding, in addition to the lateral movement in contrast to the pattern seen in most theropods but similar to that proposed for *Diplodocus* [43].

M. transversospinalis capitis, and, probably to a lesser extent, m. complexus would have contributed to dorsiflexion [2]. As the insertion point for m. transversospinalis capitis is reduced in *Nothronychus* from those observed in *Falcarius*, dorsiflexion in the former was probably correspondingly weaker. These muscles may have served as support for a small skull and stabilizers for the ventroflexive muscles, but a reduction in inertial feeding, in contrast to the hypothesized model for tyrannosaurs [32]. In many, but not all respects, *Falcarius* was a more generalized theropod than *Nothronychus* in the basicranial region, but ventroflexion is increased in both. In all therizinosaurs, this rearrangement was probably accompanied by a reduction in bite force from obligate carnivorous theropods [8].

In contrast to *Allosaurus*, the therizinosaurs exhibit a lack of well-developed ball-and-socket joints between the centra [11–13, 41]. The cervical vertebrae are not strongly opisthocoelous, nor is there a particularly spherical occipital condyle in either *Falcarius* or *Nothronychus*. Ten cervical vertebrae are well preserved in the recently described basal therizinosaur *Jianchangosaurus* [19]. Here, the centra are amphicoelous and become more elongate posteriorly. The neural arches are similar to other therizinosaurs and oviraptorosaurs [23, 42]. The occipital condyles and intervertebral centra of *Falcarius* and *Nothroncyhus* [11, 12] appear more similar to those of the tyrannosaurids [34] than allosaurs [41]. These characters might be correlated with relatively immobile joints in therizinosaurs, especially at the atlanto-occiptal articulation, between individual vertebrae in contrast to allosaurids. However, this interpretation is heavily influenced by the absence of any preserved soft tissue. Ostrich cervical vertebrae are capped by roughly 2 millimeters of cartilage [44]. As musculature and cartilages are restored, it reduces cervical flexibility in modern ostriches [45]. Avian necks have been subdivided into at least three functional regions, a slightly flexible anterior, highly mobile middle region, and a stiffer posterior portion [44–46]. In contrast to increasingly elongate cervical centra of *Jianchangosaurus*, in *Neimongosaurus*, the anteriormost centra (C2–4) are short, the middle cervical centra are long (C5–11), and the posterior most centra are very short (C12–14) [17]. The middle region comprises about 63% of the neck. Ostriches exhibit a similar pattern to *Neimongosaurus* [44]. Dzemski and Christian [44] state that complex movement, but little angular flexion, is observed in the anteriormost region of the neck in ostriches. Lateroflexion is almost inversely related to dorsoventral flexion, so that in the base of the neck, lateroflexion is at a maximum, but dorsoventral flexion is reduced. Based on similar individual cervical morphology and topologies, therizinosaurs may have been similar. Cobley et al. [46] found little apparent movement between individual vertebral centra, so total neck flexion would have to result from the summation of intervertebral movement of all of the cervical vertebrae. As a result, neck flexibility would have to be a function of the relative and absolute lengths of the constituent vertebrae. In *Neimongosaurus*, the longest cervical vertebrae are about 9% of the total neck length [17]. Therefore, total neck movement would be a function of the combined effect of all of the cervical vertebrae, rather than a high degree of freedom between any two centra in these therizinosaurs. Dzemski and Christian [44] correlate the described neck pattern with feeding at a constant level in ostriches and low grazing in sauropods such as *Diplodocus*, as also proposed by Stevens and Parrish [47]. This strategy may have been employed by therizinosaurs as well.

The occipital condyle is rounder in *Falcarius* than *Nothronychus*, but in neither case is the condyle as spherical as in other dinosaurs. Conversely, the forelimb of *Falcarius* is described as possessing an increased dorsal reach at the shoulder and dorsoventral flexibility at the wrist over the plesiomorphic theropod condition such as modeled for *Deinonychus* [48]. Therefore, food-gathering in therizinosaurs probably emphasized the arms as suggested by Zanno [49], at least as much as the neck. Such an arm versus neck issue may be more completely resolved using range of motion studies, especially at the elbow [48–51]. Such range of motion studies in the arms and pectoral girdles of therizinosaurs are hopefully forthcoming.
